# Supplementary material for: Chromosome-Level Assemblies of Three Candidatus Liberibacter solanacearum Vectors: Dyspersa apicalis (Förster, 1848), Dyspersa pallida (Burckhardt, 1986), and Trioza urticae (Linnaeus, 1758) (Hemiptera: Psylloidea)
Source: Genome Biol Evol. 2025 Jun 5;17(6):evaf116. doi: 10.1093/gbe/evaf116 (PMC12203342; doi:10.1093/gbe/evaf116)
Supplement: evaf116_Supplementary_Data [file evaf116_supplementary_data.zip › Heaven_etal_Suppl_Figs_and_Legends.pdf]

Supplementary Materials for

Chromosome-Level Assemblies of Three *Candidatus* Liberibacter solanacearum Vectors: *Dyspersa apicalis* (Förster, 1848)*, Dyspersa pallida* (Burckhardt, 1986)*,* and *Trioza urticae* (Linnaeus, 1758) (Hemiptera: Psylloidea)

Thomas Heaven[1], Thomas C. Mathers[2], Sam T. Mugford[1], Anna Jordan[1], Christa Lethmayer[3], Anne I. Nissinen[4], Lars-Arne Høgetveit[5], Fiona Highet[6], Victor Soria-Carrasco[1], Jason Sumner-Kalkun[6,^], Jay K. Goldberg[1], and Saskia A. Hogenhout[1,*].

*Correspondence: saskia.hogenhout@jic.ac.uk

This PDF file includes:

Figs. S1 to S14

Other Supplementary Materials for this manuscript include the following:

Supplementary tables S1-S6 (attached as an excell file).

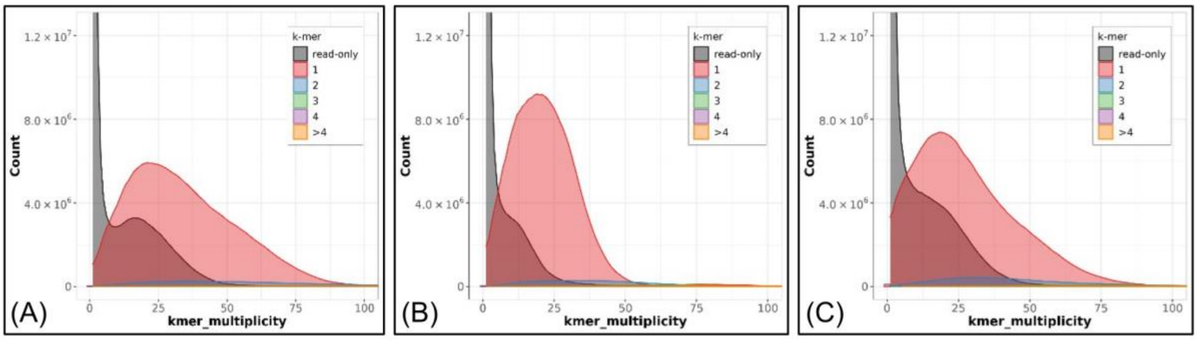

Figure S1 – *K*-mer spectra plots support minimal duplication of assemblies. K-mer plots are shown for *D. apicalis* **(A)**, *D. pallida* **(B)** and *T. urticae* **(C)**. Gray shaded areas represent *k*-mers present in sequencing reads but not an assembly whilst red shaded areas represent *k*-mers found once in an assembly, other colours represent *k*-mers found multiple times in an assembly.

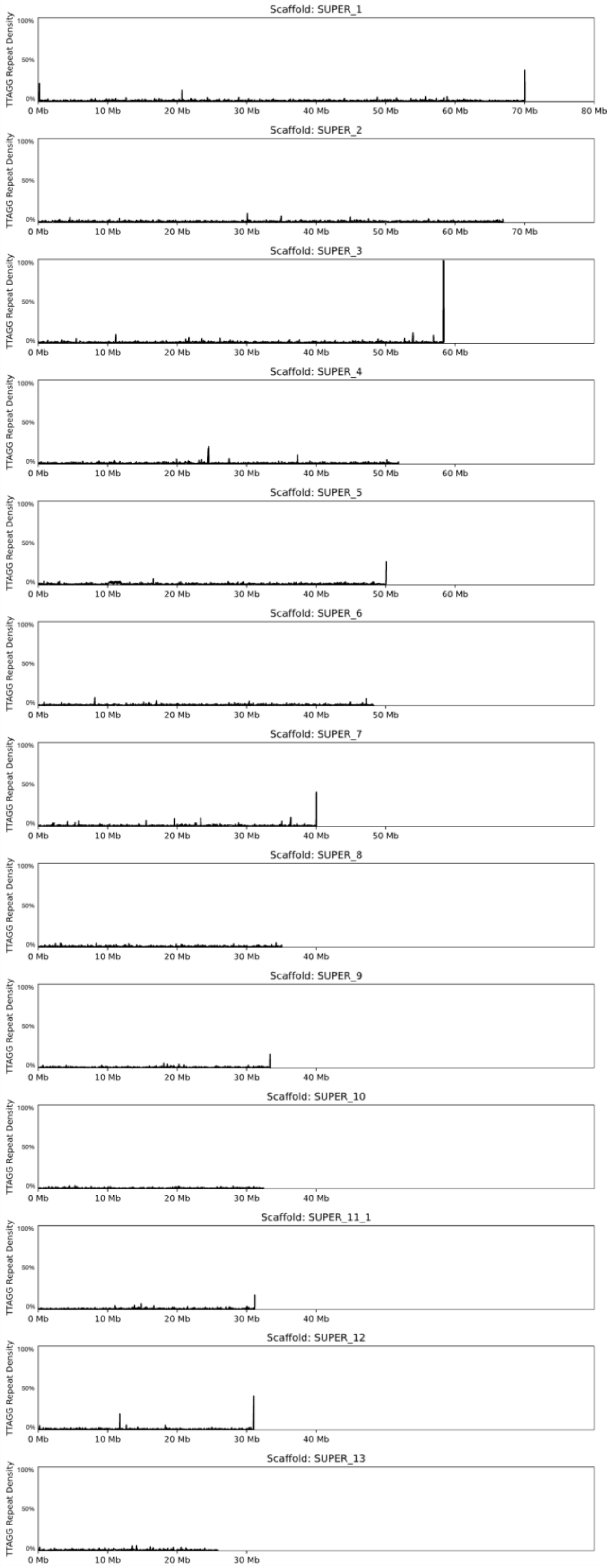

Figure S2 Occurrence of the REPEAT "TTAGG" across the thirteen largest pseudomolecules (chromosomes) of the *Dyspersa apicalis* genome. The x-axis represents the position along each pseudomolecule, while the y-axis denotes the repeat density. Where peaks are found at the ends of scaffolds (super-scaffolds 1, 3, 5, 7, 9, and 12) this indicates the presence of a telomere flanking the assembled sequence.

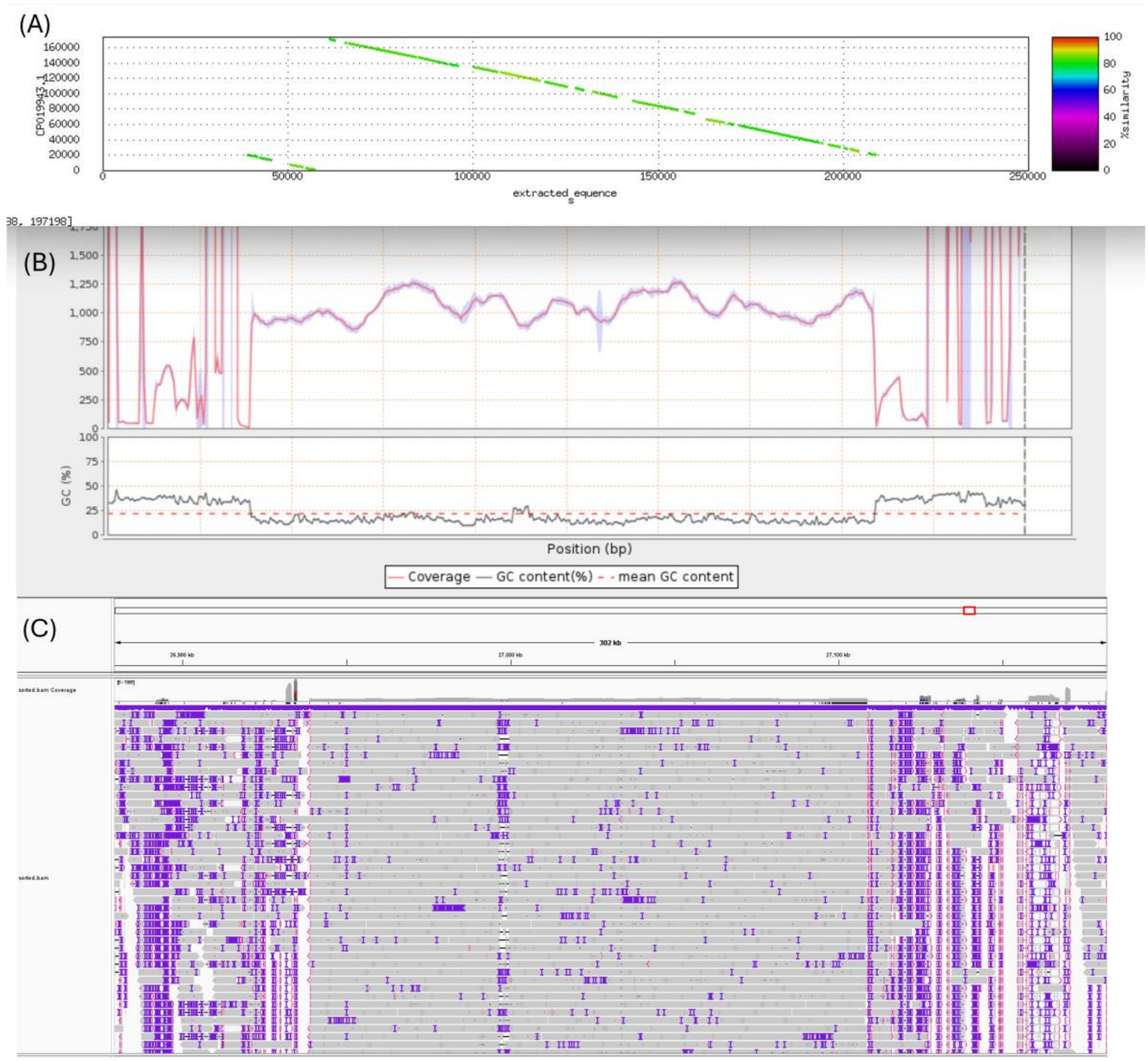

Figure S3 – Misassembled *D. apicalis* / *Ca*. C. ruddii sequence. A region of *D. apicalis* assembly with blast hits to the genome of *Ca*. C. ruddii was extracted and aligned to the *Ca*. C. ruddii reference genome (CP019943.1). An approximately 170 kb region had collinearity to the *Ca*. C. ruddii genome **(A)** has increased coverage **(B)**, and as seen in the IGV view of "sorted.bam" track is flanked by zero and one × coverage positions **(C)**.

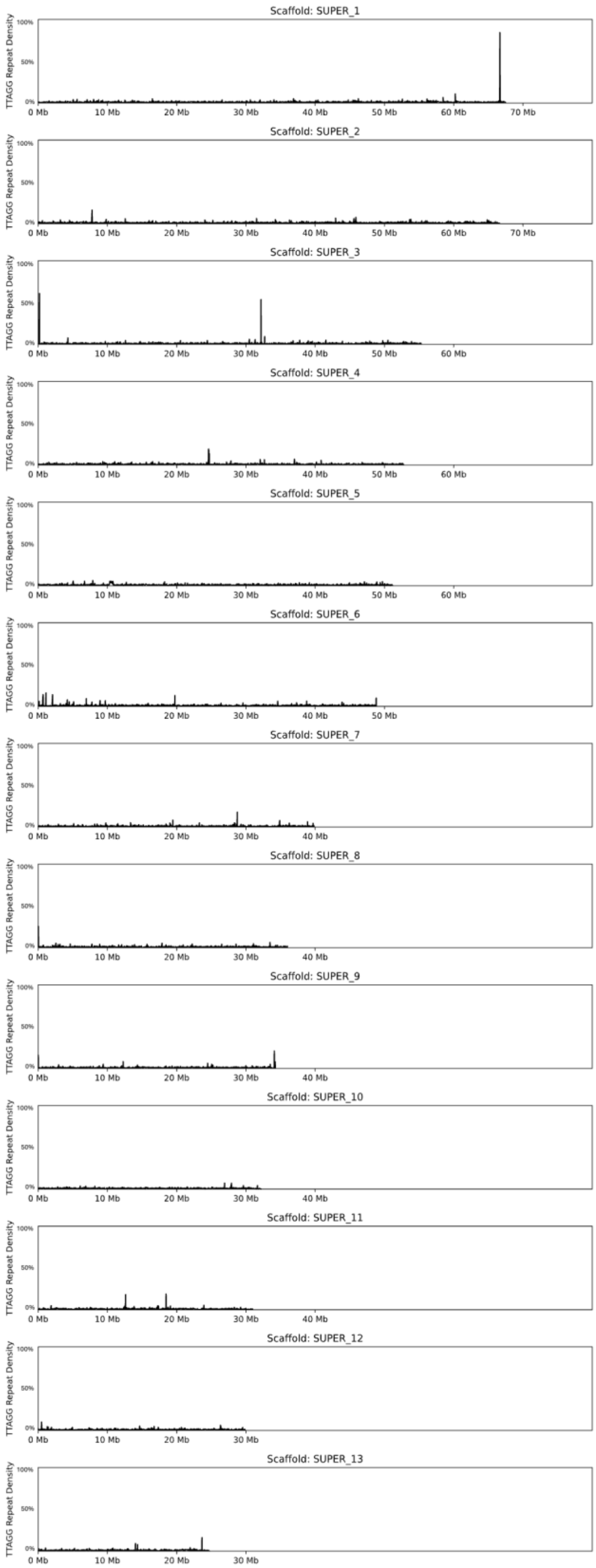

Figure S4 Occurrence of the REPEAT "TTAGG" across the thirteen largest pseudomolecules (chromosomes) of the *Dyspersa pallida* genome. The x-axis represents the position along each pseudomolecule, while the y-axis denotes the repeat density. Where peaks are found at the ends of scaffolds this indicates the presence of a telomere flanking the assembled sequence.

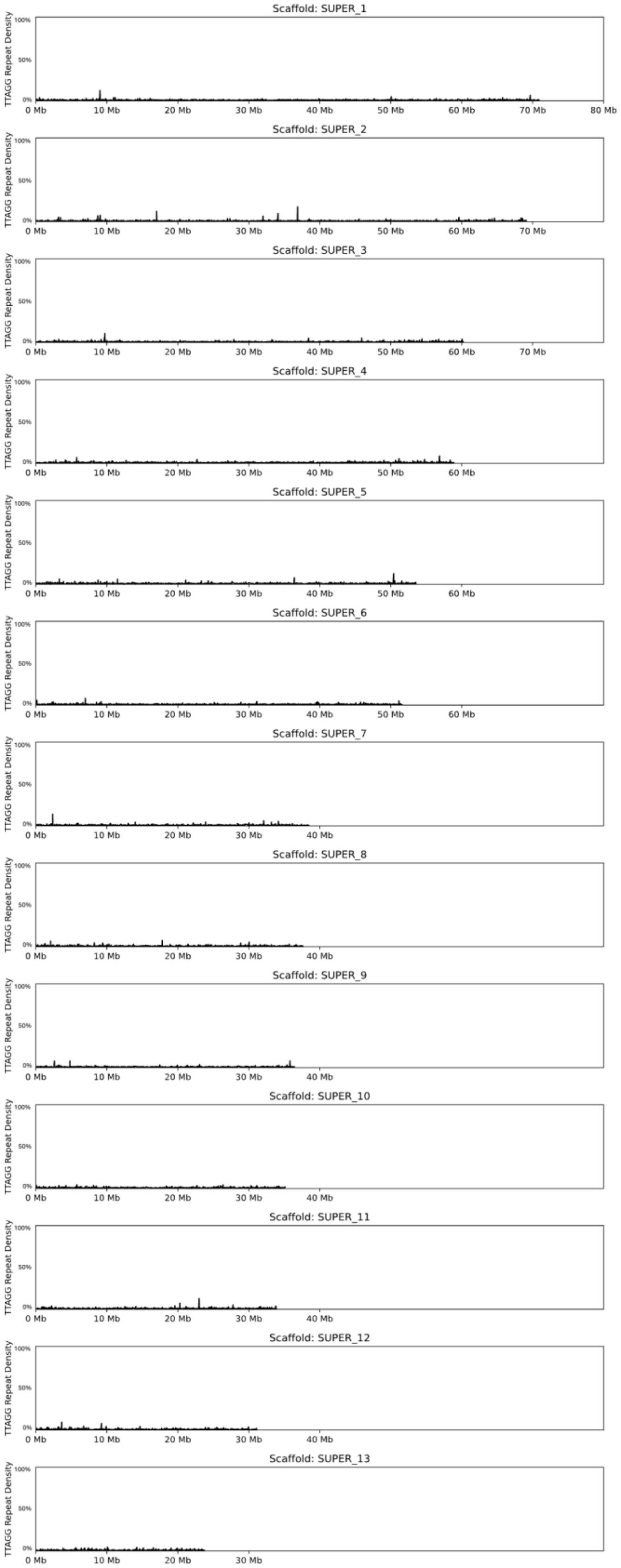

Figure S5 Occurrence of the REPEAT "TTAGG" across the thirteen largest pseudomolecules (chromosomes) of the *Trioza uritace* genome. The x-axis represents the position along each pseudomolecule, while the y-axis denotes the repeat density. There are no peaks at the ends of the scaffolds, indicating that telomere have not assembled.

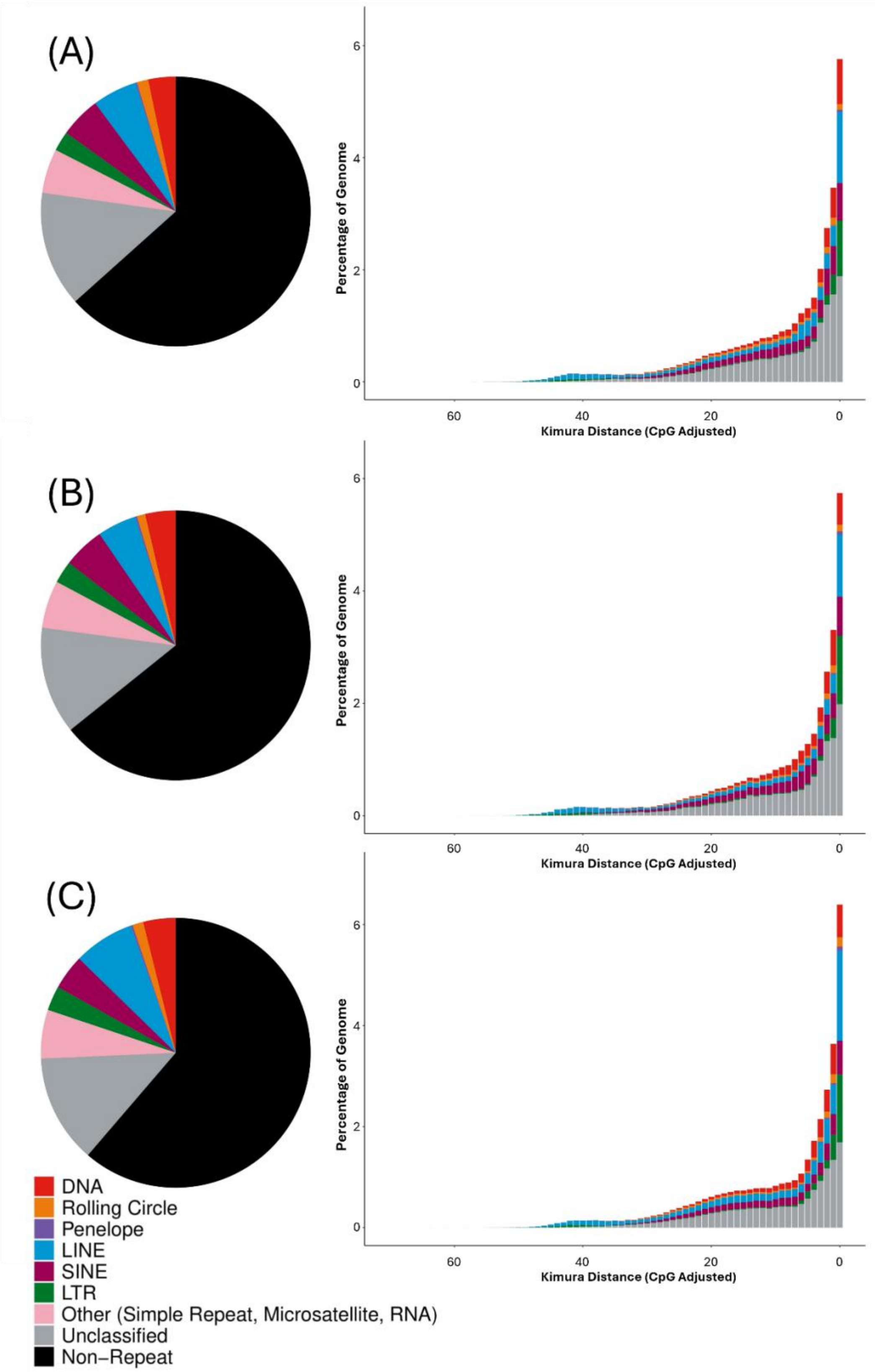

Figure S6 - Transposable element content of *Diaphorina citri* assemblies GCA_024506325.2 **(A)**, GCA_024506315.2 **(B)**,and GCA_030643865.1 **(C)**. Pie charts show TE content, different colours representing different TE superfamilies. Paired Kimura distance plots indicate the relative activity of different TE superfamilies in the genome.

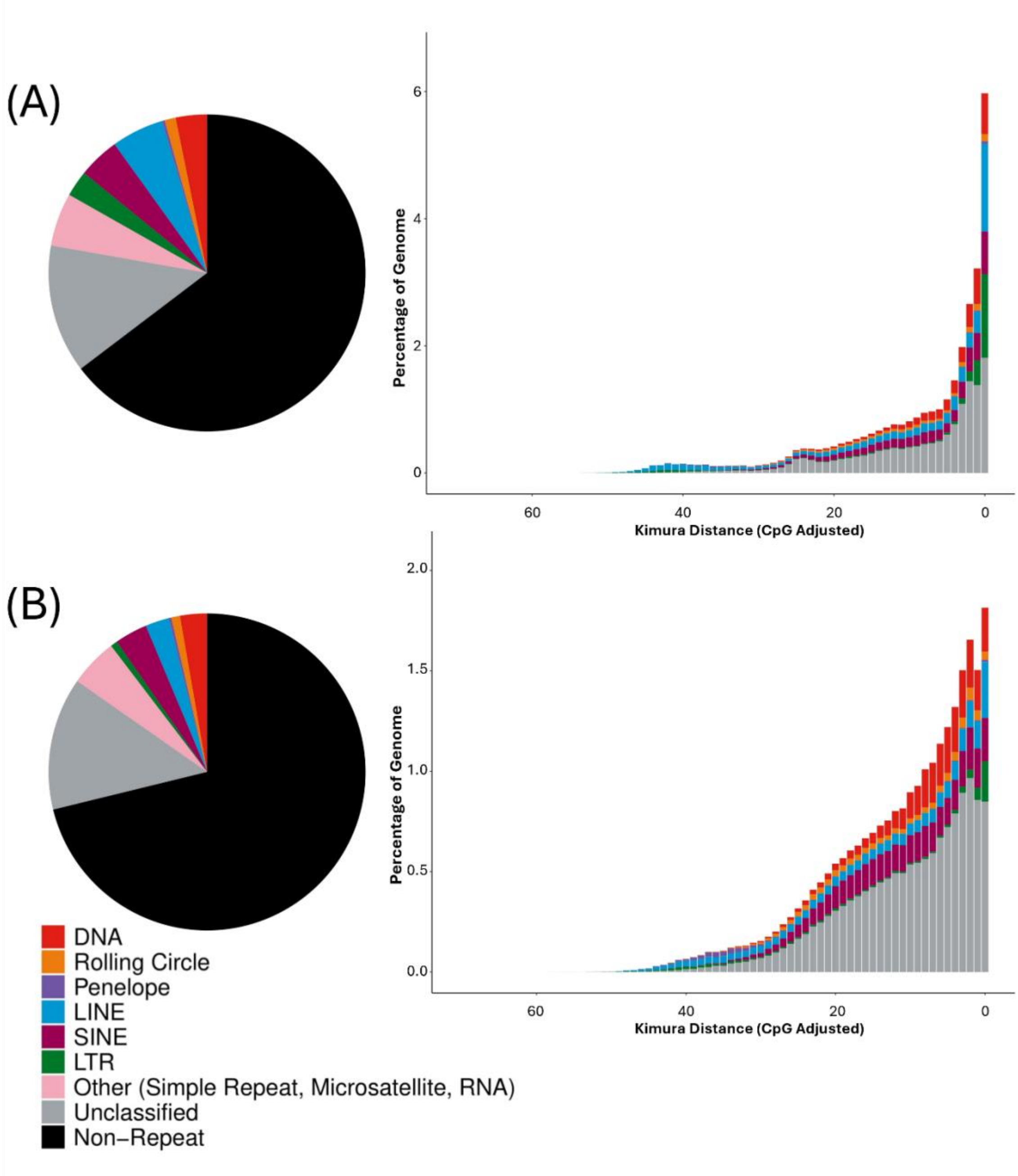

Figure S7 - Transposable element content of additional *Diaphorina citri* assemblies GCA_024506275.2 **(A)** and GCA_000475195.1 **(B)**. Pie charts show TE content, different colours representing different TE superfamilies. Paired Kimura distance plots indicate the relative activity of different TE superfamilies in the genome.

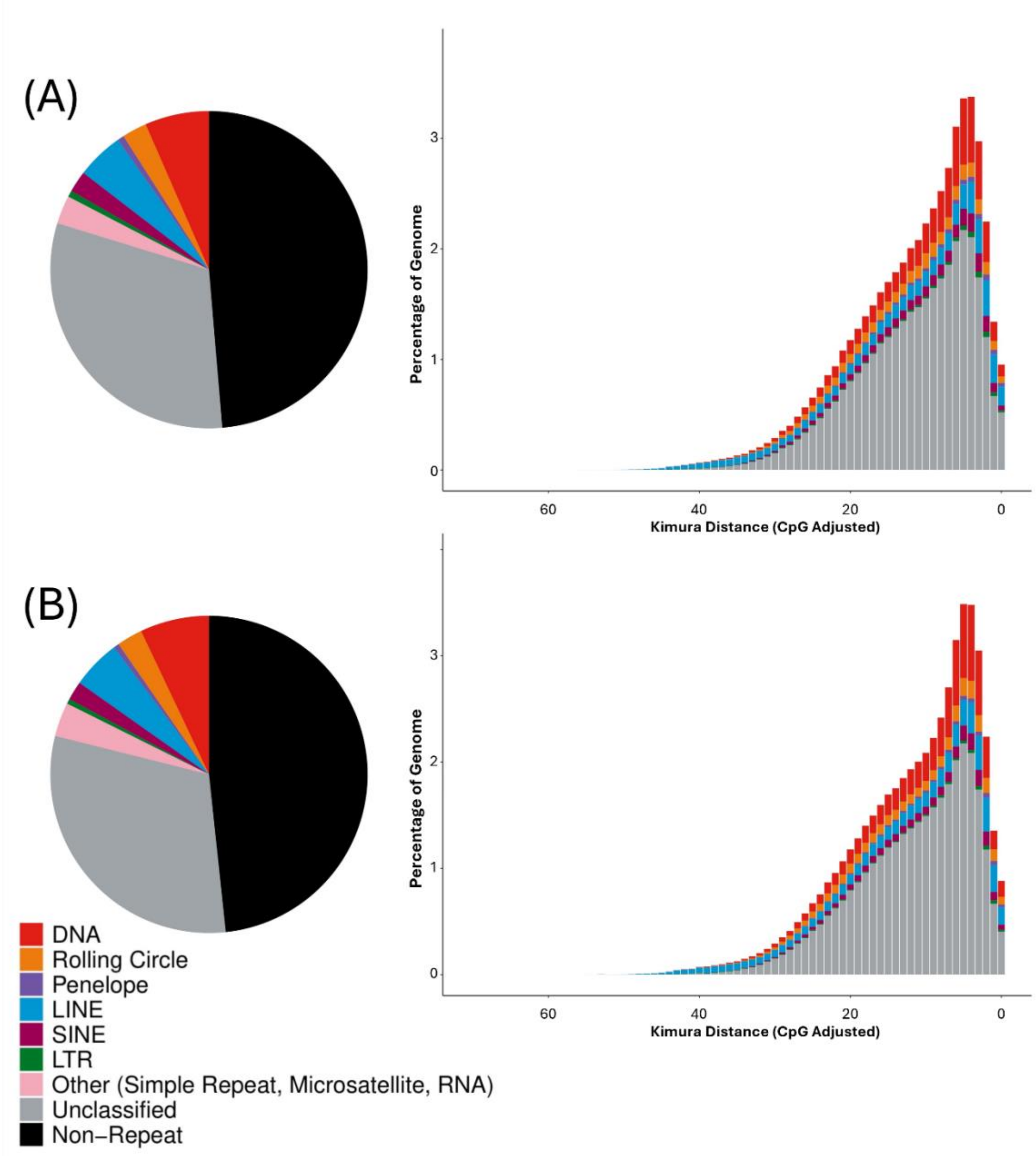

Figure S8 - Transposable element content of *Pachypsylla venusta* assemblies GCA_-12654025.1 **(A)** and GCA_000695645.2 **(B)**. Pie charts show TE content, different colours representing different TE superfamilies. Paired Kimura distance plots indicate the relative activity of different TE superfamilies in the genome.

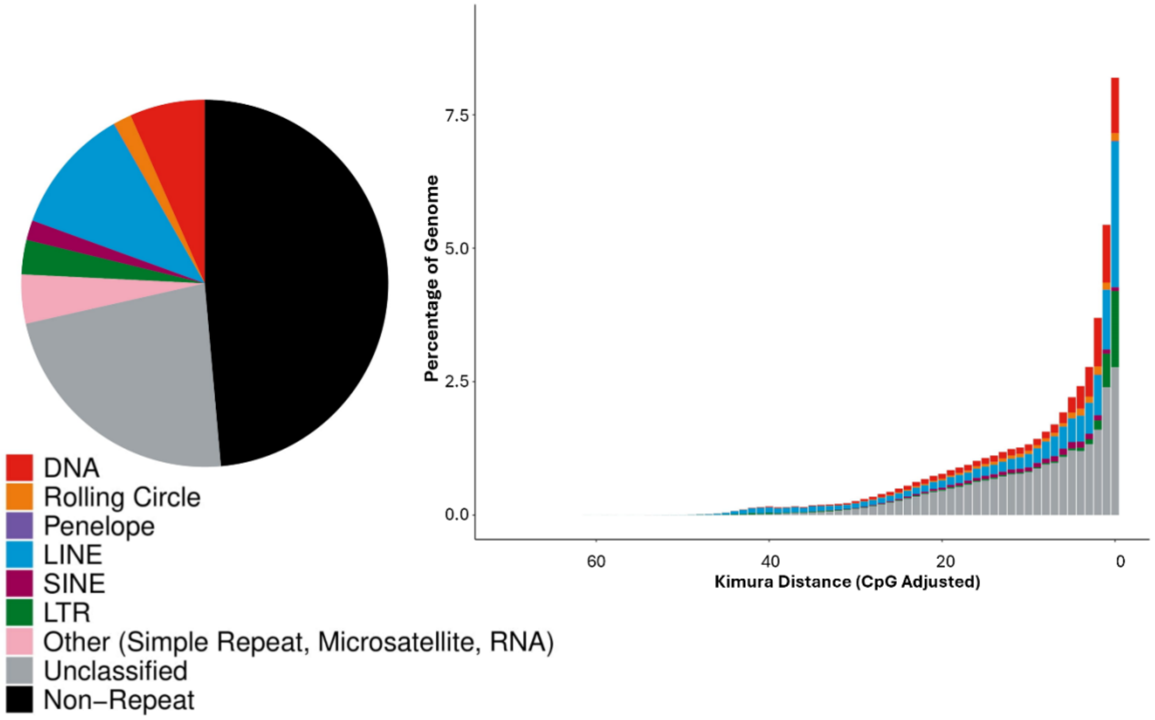

Figure S9 - Transposable element content of *Bactericera cockerelli* assembly GCA_024516035.1. Pie charts show TE content, different colours representing different TE superfamilies. Paired Kimura distance plots indicate the relative activity of different TE superfamilies in the genome.

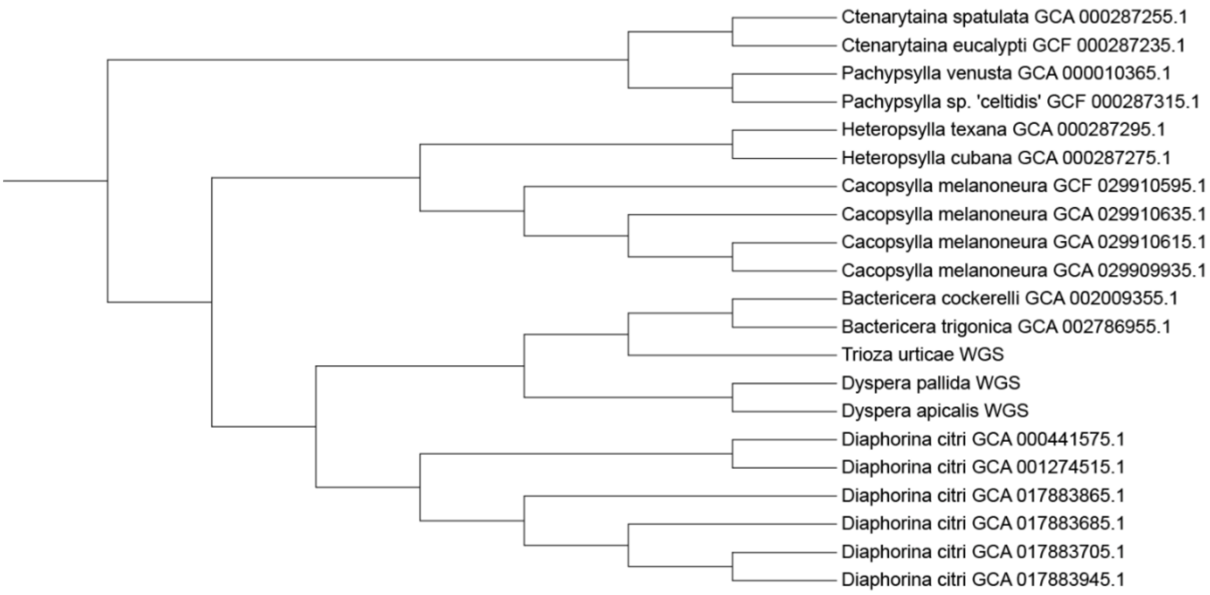

Figure S10 – Phylogeny of *Candidatus* Carsonella ruddii samples from different psyllid hosts. Downloaded genomes are identified by their host species and GenBank accession ID, *Ca.* C. ruddii sequences generated as part of this study are also included.

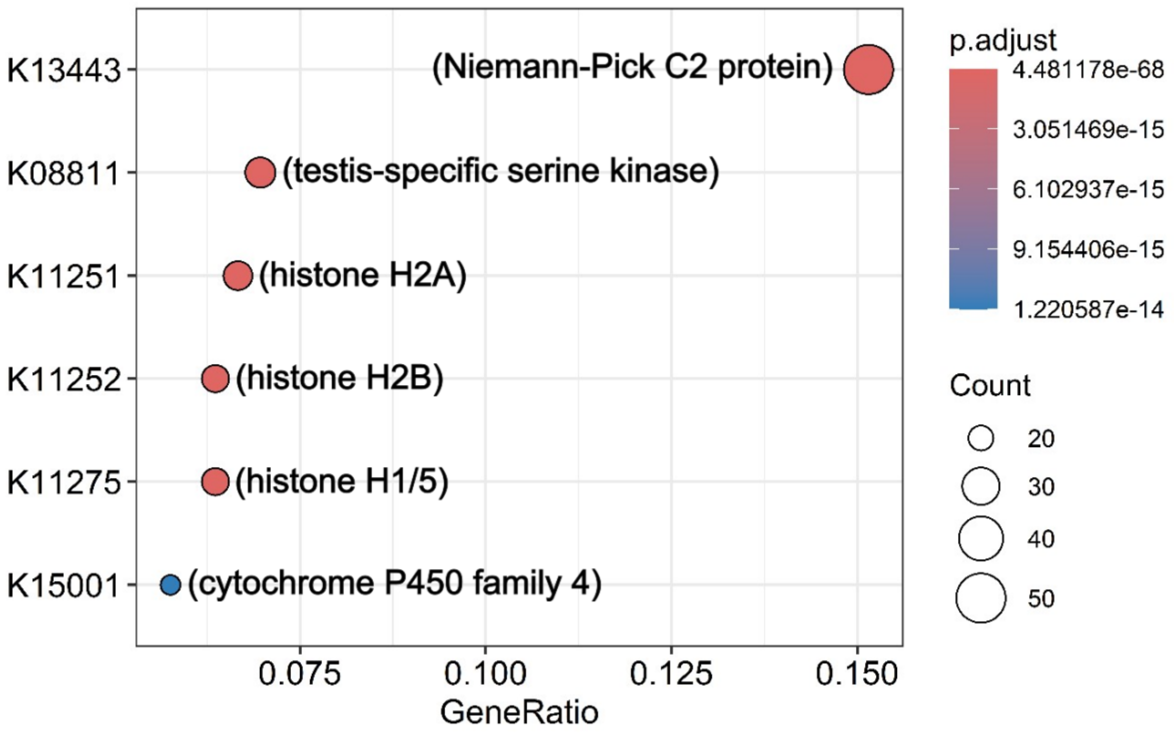

Figure S11 –Enriched KEGG terms in significantly expanded *Dyspersa apicalis* orthogroups.

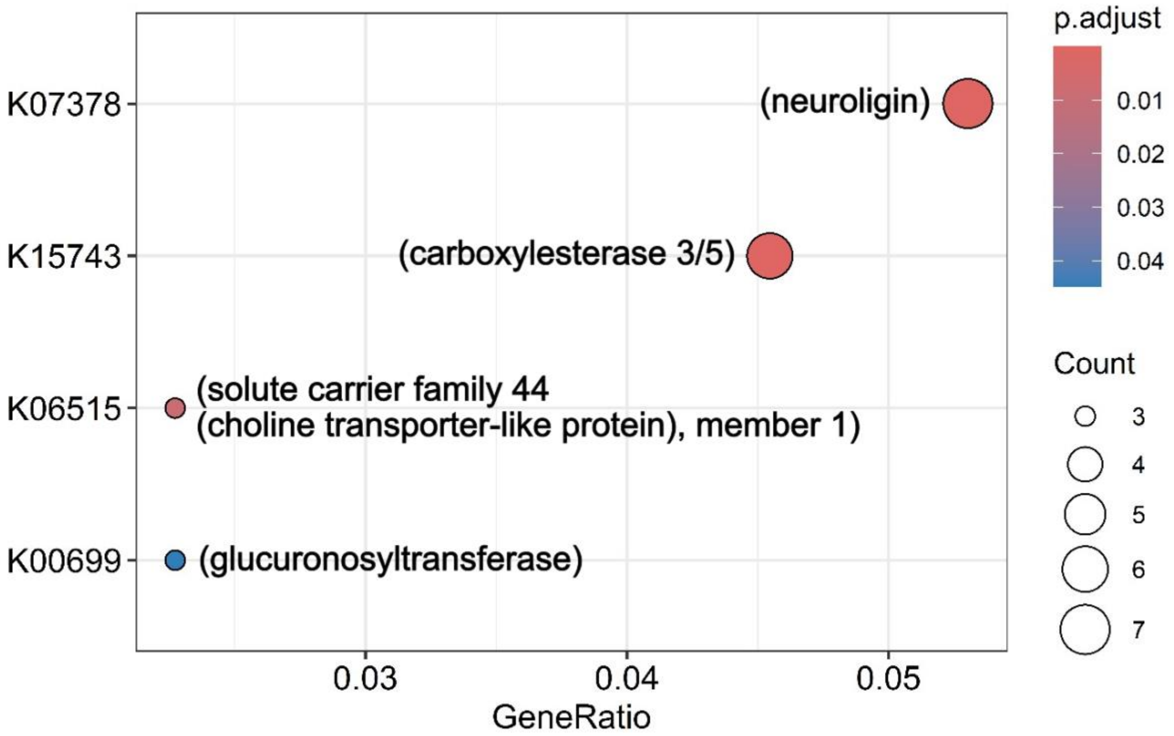

Figure S12 –Enriched KEGG terms in significantly expanded *Dyspersa pallida* orthogroups.

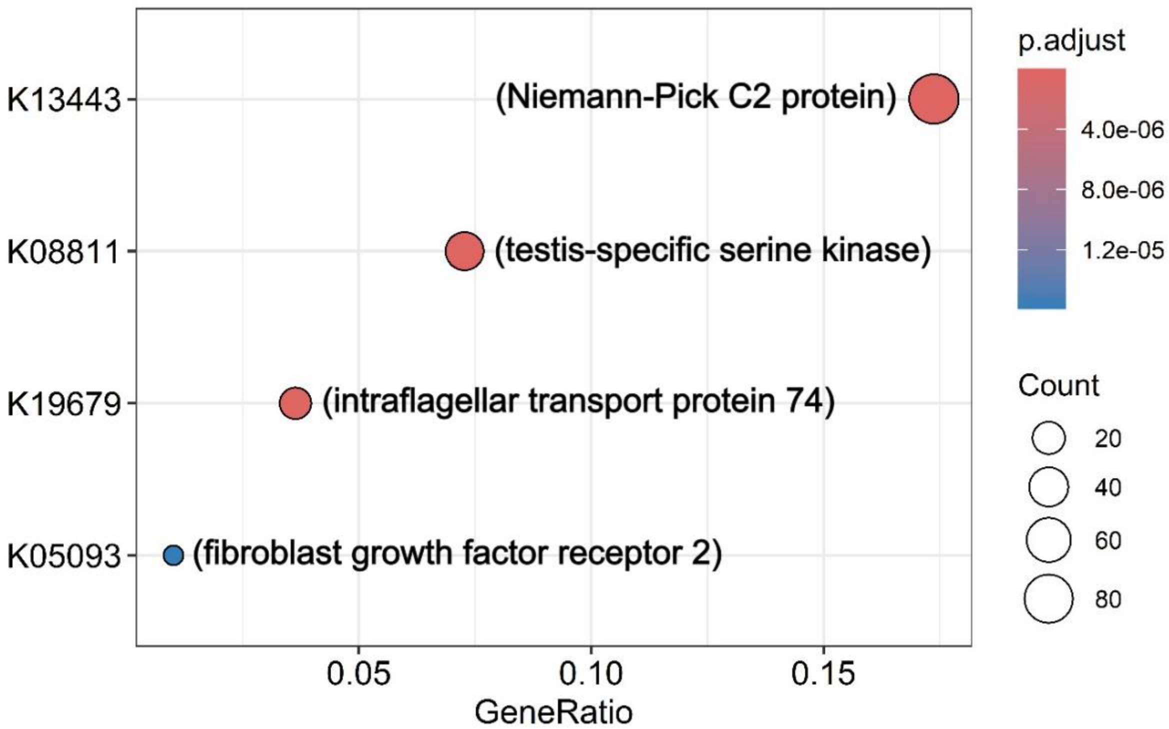

Figure S13 –Enriched KEGG terms in significantly expanded *Dyspersa* orthogroups.

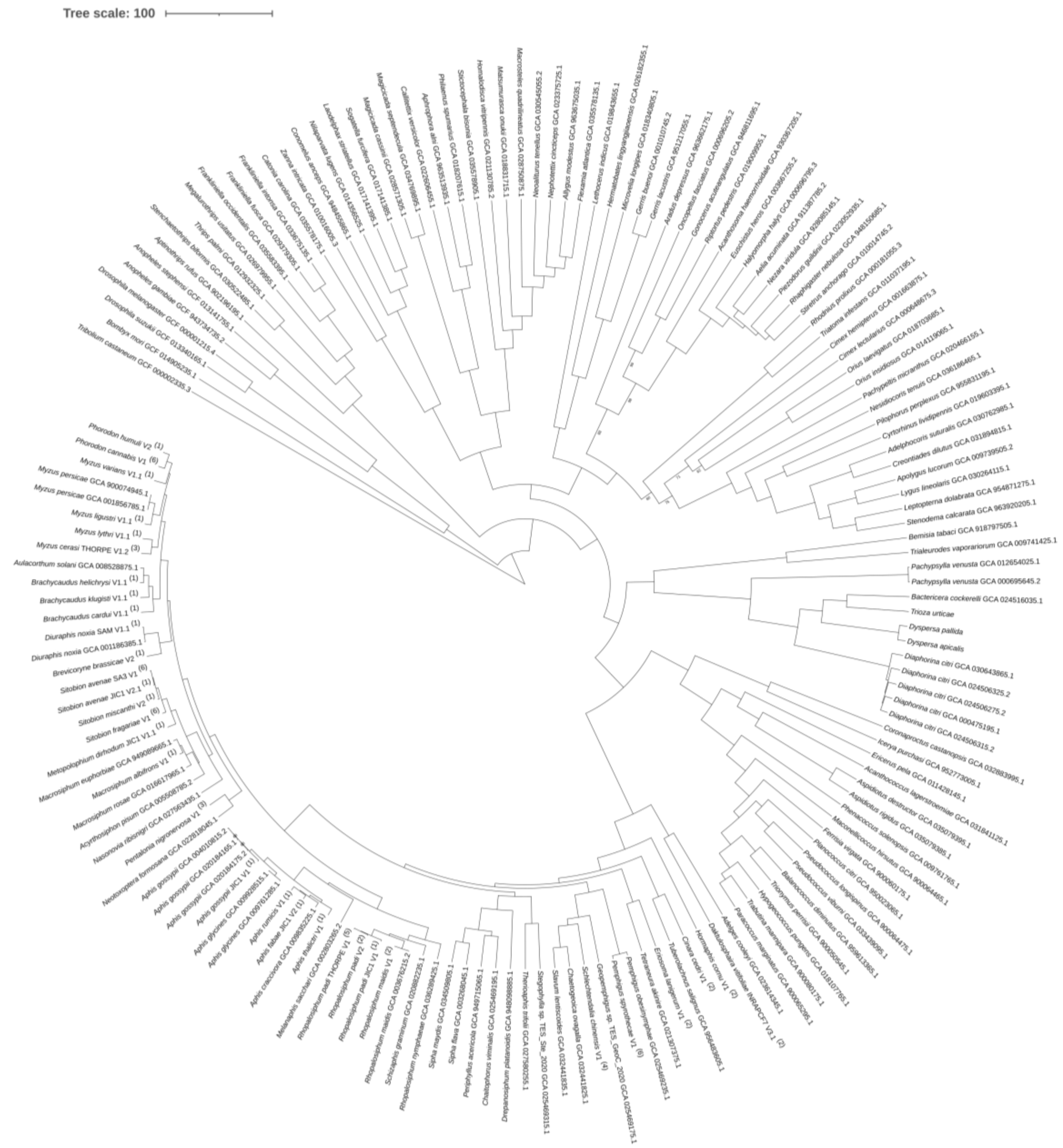

Figure S14 –Phylogeny of Hemiptera. A maximum likelihood phylogeny of available Hemiptera genomes and outgroup insects is presented (2,510 orthologues). Bootstrap values are shown for nodes with support <100. Where assemblies were accessed via the NCBI database accession numbers are given, otherwise additional genomes were sourced from: (2) aphidbase (https://bipaa.genouest.org/is/aphidbase/); zenodo archives (1) https://zenodo.org/records/5908005, (3) https://zenodo.org/records/3765644, (4) https://zenodo.org/records/5511862), and (5) https://zenodo.org/records/1252934; and (6) unpublished assemblies shared with the lab.
